# Harveian Society

**Published:** 1897-04-10

**Authors:** 


### Medical Progress and Hospital Clinics.

[*The Editor will be glad to receive offers of co-operation and contributions from members of the profession. All letters should be addressed to* The Editor, at the Office, 28 & 29, Southampton Street, Strand, London, W.C.]

### HARVEIAN SOCIETY.

At the meeting held on April 1st, Mr. Buckston Browne read a paper on the question "Is it ever Impossible to Pass a Catheter Through the Urethra into the Bladder?" He maintained that it was never impossible to pass an instrument, even in the worst cases of stricture of the urethra, unless the urethra had in some part of its course actually ceased to exist. If an instrument was once passed the case could be brought to a successful issue without any perineal incision, and, that being so, the patient was saved all the risks of hæmorrhage, and of that terrible misfortune a perineal fistula. In the most severe cases of prostatic enlargement the urethra was simply tortuous. The difficulties were fully described, and instruments were shown by which they could all be overcome. In no prostatic case was it allowed that the urethra was impassable by instruments, and therefore there was rarely any real need for any form of prostatectomy, or for castration. All the other forms of urethral obstruction were discussed, and the question which formed the title of the paper was answered emphatically in the negative.

